# Uncovering the dispersion history, adaptive evolution and selection of wheat in China

**DOI:** 10.1111/pbi.12770

**Published:** 2017-07-17

**Authors:** Yong Zhou, Zhongxu Chen, Mengping Cheng, Jian Chen, Tingting Zhu, Rui Wang, Yaxi Liu, Pengfei Qi, Guoyue Chen, Qiantao Jiang, Yuming Wei, Ming‐Cheng Luo, Eviatar Nevo, Robin G. Allaby, Dengcai Liu, Jirui Wang, Jan Dvorák, Youliang Zheng

**Affiliations:** ^1^ Triticeae Research Institute Sichuan Agricultural University Chengdu Sichuan China; ^2^ Chengdu City Institute of Archaeology Chengdu Sichuan China; ^3^ Department of Plant Sciences University of California Davis CA USA; ^4^ State Key Lab of CAD&CG Zhejiang University Hangzhou Zhejiang China; ^5^ Institute of Evolution University of Haifa Haifa Israel; ^6^ School of Life Sciences University of Warwick Coventry UK; ^7^ Ministry of Education Key Laboratory for Crop Genetic Resources and Improvement in Southwest China Sichuan Agricultural University Yaan Sichuan China

**Keywords:** wheat, landrace, dispersion, adaption, selection

## Abstract

Wheat was introduced to China approximately 4500 years ago, where it adapted over a span of time to various environments in agro‐ecological growing zones. We investigated 717 Chinese and 14 Iranian/Turkish geographically diverse, locally adapted wheat landraces with 27 933 DArTseq (for 717 landraces) and 312 831 Wheat660K (for a subset of 285 landraces) markers. This study highlights the adaptive evolutionary history of wheat cultivation in China. Environmental stresses and independent selection efforts have resulted in considerable genome‐wide divergence at the population level in Chinese wheat landraces. In total, 148 regions of the wheat genome show signs of selection in at least one geographic area. Our data show adaptive events across geographic areas, from the xeric northwest to the mesic south, along and among homoeologous chromosomes, with fewer variations in the D genome than in the A and B genomes. Multiple variations in interdependent functional genes such as regulatory and metabolic genes controlling germination and flowering time were characterized, showing clear allelic frequency changes corresponding to the dispersion of wheat in China. Population structure and selection data reveal that Chinese wheat spread from the northwestern Caspian Sea region to South China, adapting during its agricultural trajectory to increasingly mesic and warm climatic areas.

## Introduction

Bread (common) wheat (*Triticum aestivum*, genomes AABBDD) originated approximately 8000 years ago (Nesbitt and Samuel, 1996; Willcox, 1997) by hybridization of tetraploid *Triticum turgidum* (genomes AABB) with diploid *Aegilops tauschii* (genomes DD) (Kihara, 1944; McFadden and Sears, 1966). Genetic evidence points to the southwestern coastal area of the Caspian Sea as the geographic origin of bread wheat (Wang *et al*., 2013). The westward spread of bread wheat cultivation from this area resulted in sympatry with both cultivated tetraploid wheat, which was at that time the dominant crop in West Asia, and wild tetraploid emmer. Sympatry in this geographic area resulted in gene flow from tetraploid wheat to the bread wheat A and B genomes (Akhunov *et al*., 2010; Dvorak *et al*., 2006). Gene flow between the two genomes then became limited as bread wheat cultivation moved eastwards, away from the centre of tetraploid wheat diversity (Dvorak *et al*., 2006; Wang *et al*., 2013). Although bread wheat continued to have sympatry with *Ae. tauschii*, which is widespread in eastern Iran, Turkmenistan and Afghanistan, as it continued its eastward trek of cultivation, this was unlikely to result in gene flow into bread wheat because of the strong reproductive barriers that exist between the two species. Different opportunities for gene flow from bread wheat progenitors to bread wheat resulted in the longitudinal subdivision of bread wheat into western and eastern populations (Balfourier *et al*., 2007; Dvorak *et al*., 2006; Tsunewaki, 1968) and led to different levels of genetic diversity in the A and B genomes when compared with the D genome (Dvorak *et al*., 2011). Limited gene flow in East Asia, such as in China, is expected to conserve the genetic structure of Chinese bread wheat more than in wheat populations elsewhere (Dvorak *et al*., 2006; Wang *et al*., 2013). Studies of genetic diversity in Chinese wheat populations may provide valuable clues about the structure of genetic diversity in the early stages of bread wheat evolution, and Chinese wheat populations may serve as a reference for the study of genetic diversity of bread wheat elsewhere.

Bread wheat cultivation reached China approximately 4500 years ago. Bread wheat remnants were found at the ‘Donghuishan’ site in Gansu and were dated to 4605 ± 150 and 4260 ± 80 BP (Before Present), respectively (Li and Wang, 2013; Zhang, 1983); they are the oldest remnants found to date. Historical records suggest that bread wheat was first cultivated in northwest China and spread from there to East China, and then to the South and southwest China (Zeng, 2005a; Zhuang, 2002). During the spread of bread wheat cultivation, wheat adapted to different environmental conditions and became one of the most successful crops in China (Zeng, 2005a). Today, China is the world's largest wheat producer.

**Figure 1:**
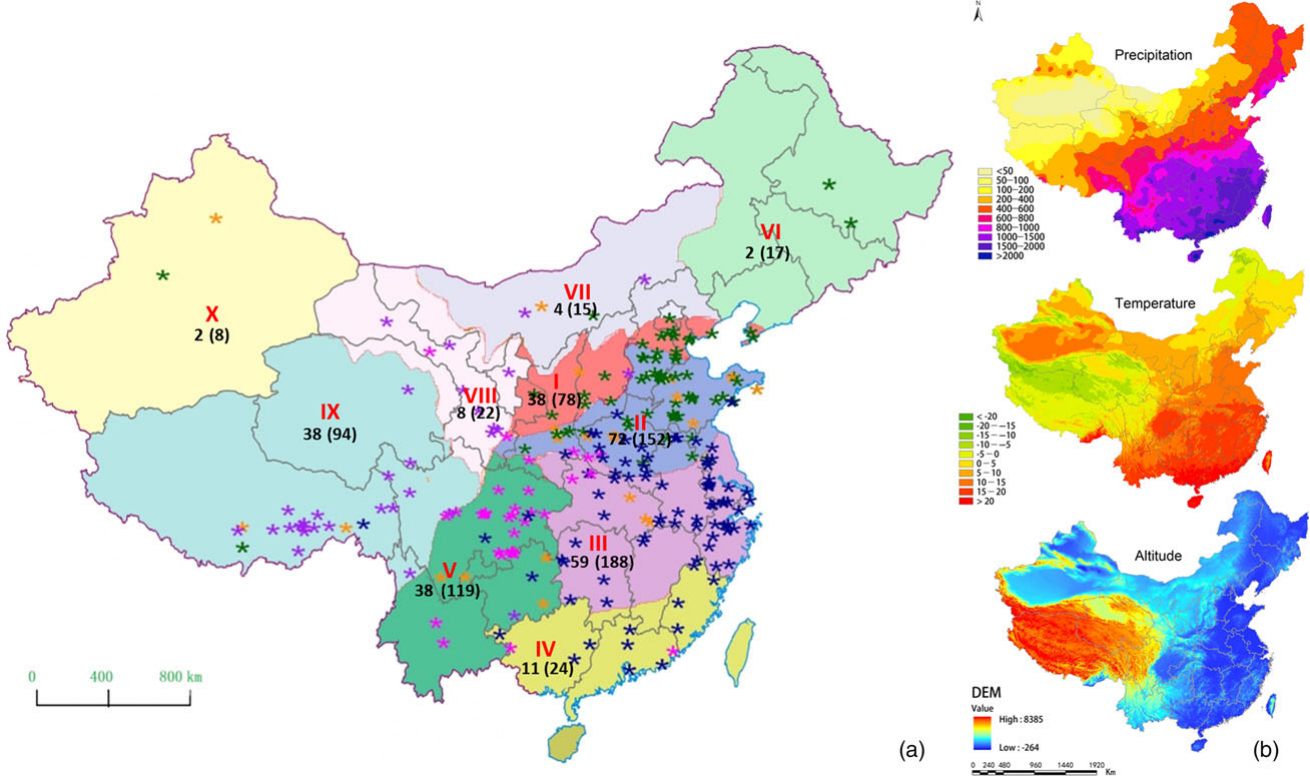
Ten agro‐ecological wheat growing zones in China (He *et al*., 2001). I‐NW (northern winter wheat zone), II‐Y&H (Yellow and Huai River valleys facultative wheat zone), III‐YTS (middle and low Yangtze valley autumn‐sown spring wheat zone), IV‐SAS (southern autumn‐sown spring wheat zone), V‐SWAS (southwestern autumn‐sown spring wheat zone), VI‐NES (northeastern spring wheat zone), VII‐NS (northern spring wheat zone), VIII‐NWS (northwestern spring wheat zone), IX‐Q&T (Qinghai–Tibetan plateau spring–winter wheat zone) and X‐XJ (xinjiang winter‐spring wheat zone). (a) The numbers of landraces genotyped by the Wheat660 SNP array and DArTseq (in parentheses) in each zone. The stars indicate the geographic locations of landraces and colour of stars indicate the genetic ancestry of landraces for *K* = 5 inferred using Bayesian classification. (b) Maps of China showing annual rainfall, annual temperature and elevation. The environment and ecological data were provided by the Data Center for Resources and Environmental Sciences, Chinese Academy of Sciences (RESDC) (http://www.resdc.cn).

Wheat is grown in China in ten agro‐ecological zones (He *et al*., 2001; Jin, 1996) that we have labelled I‐NW, II‐Y&H, III‐YTS, IV‐SAS, V‐SWAS, VI‐NES, VII‐NS, VIII‐NWS, IX‐Q&T and X‐XJ (see Figure 1). Adaption of wheat to these diverse environments led to the formation of landraces (Dwivedi *et al*., 2016), which are geographically isolated and locally adapted ecotypes (Belay *et al*., 1995; Dotlačil *et al*., 2010; Jones *et al*., 2008). These wheat landraces have a high capacity to tolerate local biotic and abiotic stresses, resulting in the high stability of their yields and but, because they are grown under a low‐input agricultural regime, they produce an intermediate yield level when compared to modern agricultural expectations for grain yield (Zeven, 1998). The conservation of these adaptive traits make wheat landraces a valuable resource for breeding locally adapted varieties for modern, high‐input agriculture (Keller *et al*., 1991; Tesemma *et al*., 1998).

In this study, the genetic structure of 717 Chinese landraces from all ten agro‐ecological zones was investigated using the Diversity Arrays Technology (DArT) and single‐nucleotide polymorphism (SNP) marker systems. The wheat landrace collection represents an invaluable resource for the analysis of micro‐evolution under natural and artificial selection. The characterization of chromosome regions showing evidence of selective sweeps and genome‐wide association analyses of important agronomic traits may lead to the discovery of economically or biologically important genes and ultimately translate into the genetic improvement of locally adapted modern wheat varieties.

## Results

### Genetic diversity of Chinese wheat landraces

After quality control, a final data set was obtained consisting of 717 landraces genotyped with 27 933 DArTseq markers (18 902 DArT and 9031 DArT_GBS markers) and 285 landraces genotyped with 312 831 SNP markers (Table S2; Figure S1). The genomic locations of these 340 764 markers were determined with the Chinese Spring survey sequence (IWGSC and TGAC).

**Figure 2:**
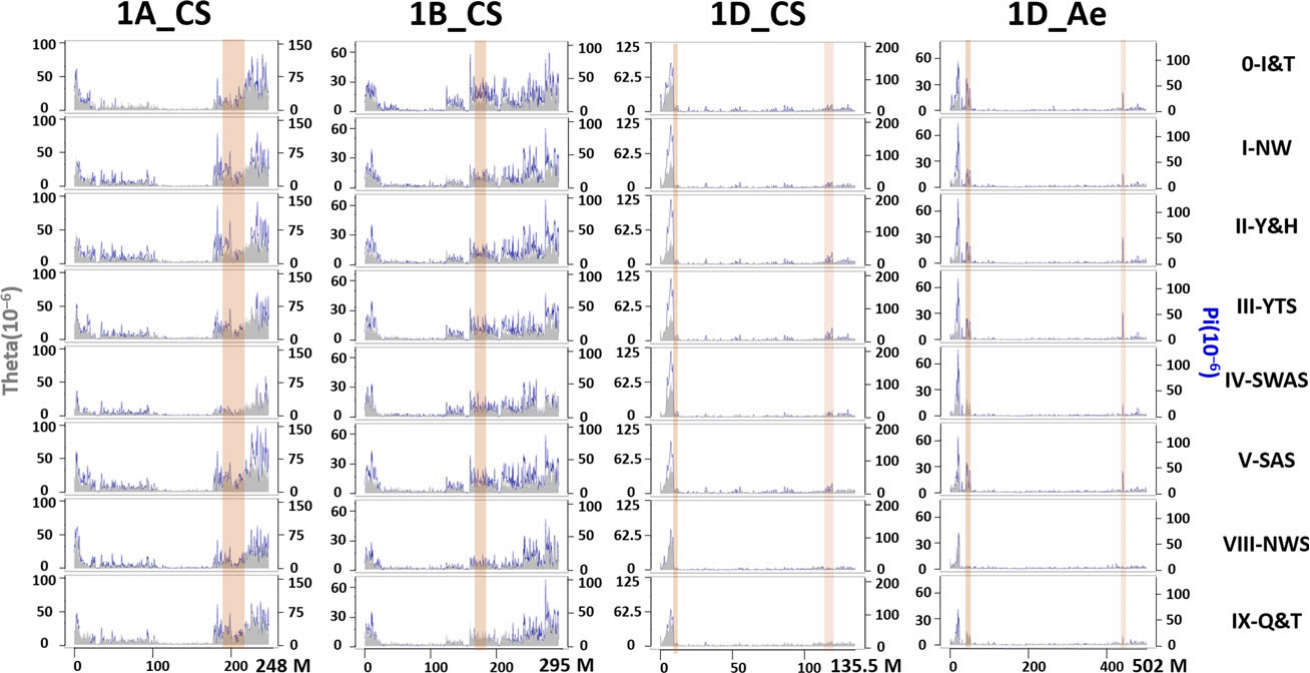
Nucleotide diversity (π, dark blue) and Watterson estimator (θ, grey) along chromosomes 1A, 1B and 1D of wheat and 1D of *Ae. tauschii* for wheat landraces in seven main areas in China and Iran/Turkey (0). Figure S5 shows the other 18 chromosomes. The details of the genetic index for each chromosome and 0.5 grids along chromosomes were listed in Table S3 and S6, respectively.

Both DArTseq and Wheat660K SNP markers showed a high polymorphism information content (PIC) (0.27 and 0.32, respectively in Table S4). The heterozygosity detected by the Wheat660K was about sixfold higher than that detected by DArTseq (0.07 and 0.01, respectively) (Table S1; Figure S2). V‐SWAS had the highest expected heterozygosity (gene diversity) in all wheat growing zones, while IV‐SAS had the lowest expected heterozygosity (Table S1). In the population of 717 landraces genotyped by DArTseq markers, the mean nucleotide diversity (π) was 0.20 × 10^−4^, the mean minor allele frequency (MAF) was 11.70%, and the mean distance between markers was 179 Kb. In the population of 285 landraces genotyped with Wheat660K SNP markers, π = 0.21 × 10^−4^, mean MAF = 12.95%, and the mean distance between markers was 16 Kb (Table S4; Figure S2). For both marker systems, genetic diversity was higher in the distal regions than in the proximal regions of most chromosomes (Figures S2, S3). Genetic diversity was consistent between the D genomes of wheat and *Ae. tauschii* for the distribution of Wheat660K SNP markers, π and Tajima's *D* along the chromosomes (Figures 2, S4, S5).

Diversity varied among landraces from different growing zones (Table S3; Figures 2, S5). The lowest was in zone IV‐SAS (π = 0.57 × 10^−4^) and the highest in its adjacent zone V‐SWAS (π = 0.97 × 10^−4^). The landraces from two neighbouring zones I‐NW and II‐Y&H in North China were least genetically differentiated (fixation index, *F*_ST_ = 0.03), while two neighbouring zones in South China, IV‐SAS and IX‐Q&T, were most genetically differentiated (*F*_ST_ = 0.36) (Table S5). Based on the negative Tajima's D values (Table S6), all of the zones contain an excessive number of rare alleles, suggesting an ongoing expansion of the population during wheat selection.

### Population structure of Chinese wheat landraces

**Figure 3:**
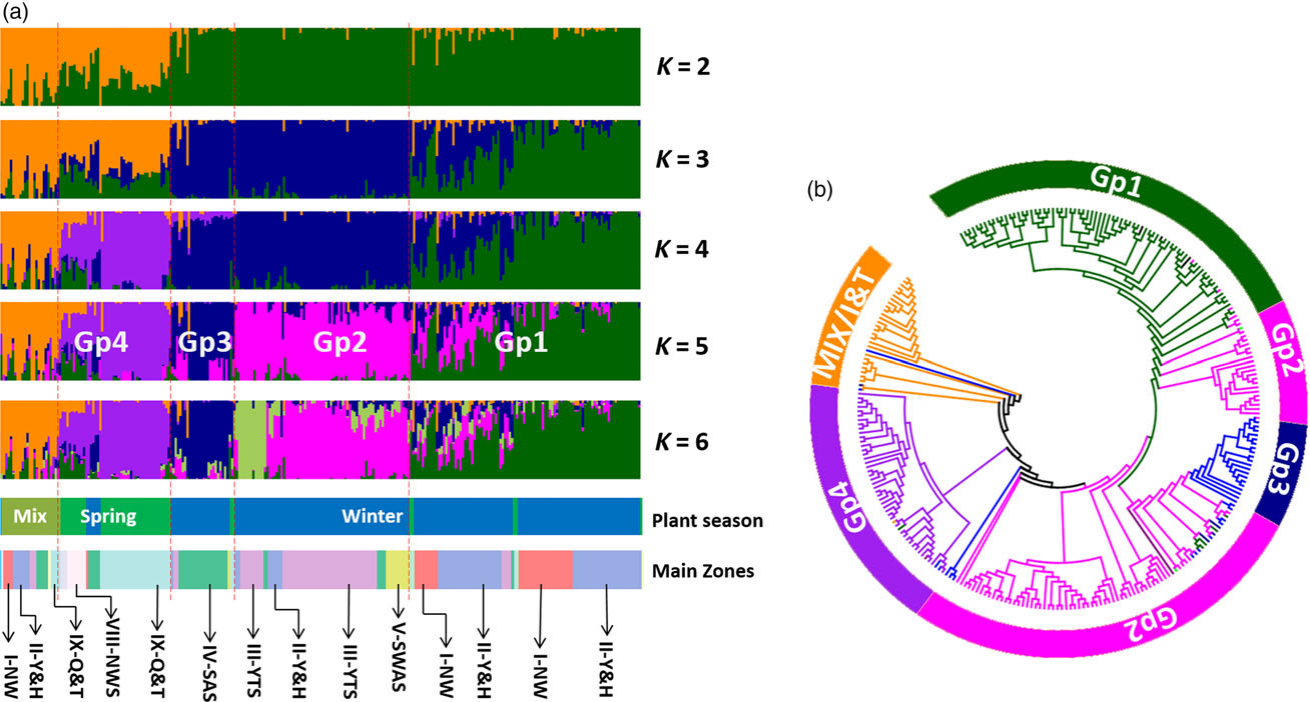
Population structure of 285 landraces including 13 landraces from Iran and Turkey computed using merged DArTseq and wheat660K array data for *K* = 2 to 6 (a). When *K* = 5, the accessions for five groups (Mix and Gp 1–4) were from the following groups: Iran/Turkey and Mix zones (Mix), I‐NW and II‐Y&H (Gp1), III‐YTS, small part of II‐Y&H and V‐SWAS (Gp2), IV‐SAS (Gp3), IX‐Q&T and VIII‐NWS (Gp4). Comparison of the neighbour‐joining tree and topological structure groups when *K* = 5 (b). The colour of each accession in the NJ tree of Fig. 3B was according to the topological structure groups in (a). The details of results based on DArT or Wheat660K SNP markers were in Fig. S6A‐S6D.

Based on the pattern and consistency of individual landrace assignments, a *K* value of 2–6 was concluded to capture most of the biologically relevant information on population structure. For these levels of *K*, landrace assignments into groups generated by DArTseq (Figure S6A), Wheat660K SNP (Figure S6B) and combined data (Figure 3a) were similar. At *K* = 2, spring wheat and Iranian and Turkish landraces (designated as Mixed) formed one cluster, and winter wheat formed the other cluster (Figure 3). At *K* = 3, winter landraces were divided into a South China cluster (III‐YTZ, IV‐SAS, V‐SWAS) and a North China cluster (I‐NW and II‐Y&H). At *K* = 4, the landraces from spring wheat zones (VIII‐NWS, IX‐Q&T and X‐XJ) were separated from the mixed group. At *K* = 5, the landraces in the winter wheat zones III‐YTZ, II‐Y&H and V‐SWAS were separated from IV‐SAS.

To investigate the phylogenetic relationships among Chinese wheat landraces, genetic distances between clusters were computed from data generated with Wheat660K SNP markers, and neighbour‐joining (NJ) trees were constructed (Figure 3b). The topologies of trees produced using either DArT, Wheat660K SNP or the combined data were consistent (Figure S6C–E). Data from Structure (*K *= 5) and the NJ trees clustered the landraces into five groups (Gp1‐Gp4 and MIX). The landraces in Gp2 were the only group in which the data from Structure showed that the landraces were clustered separately in the NJ tree (Figure 3b, Table S1). The landraces from Iran and Turkey (MIX) were grouped into a single group, which was phylogenetically close to spring wheat originating from zones X‐XJ, VII‐NS and VIII‐NWS (Figure S6D). Ignoring the MIX group, the classification of landraces into groups GP1 to GP4 closely fit environmental factors, such as annual rainfall and humidity in the zones (Figures 1b, S6F). This was confirmed by multiple linear regression analyses, which suggested that environmental factors played important roles in shaping diversity and zone divergence (Table S7).

**Figure 4:**
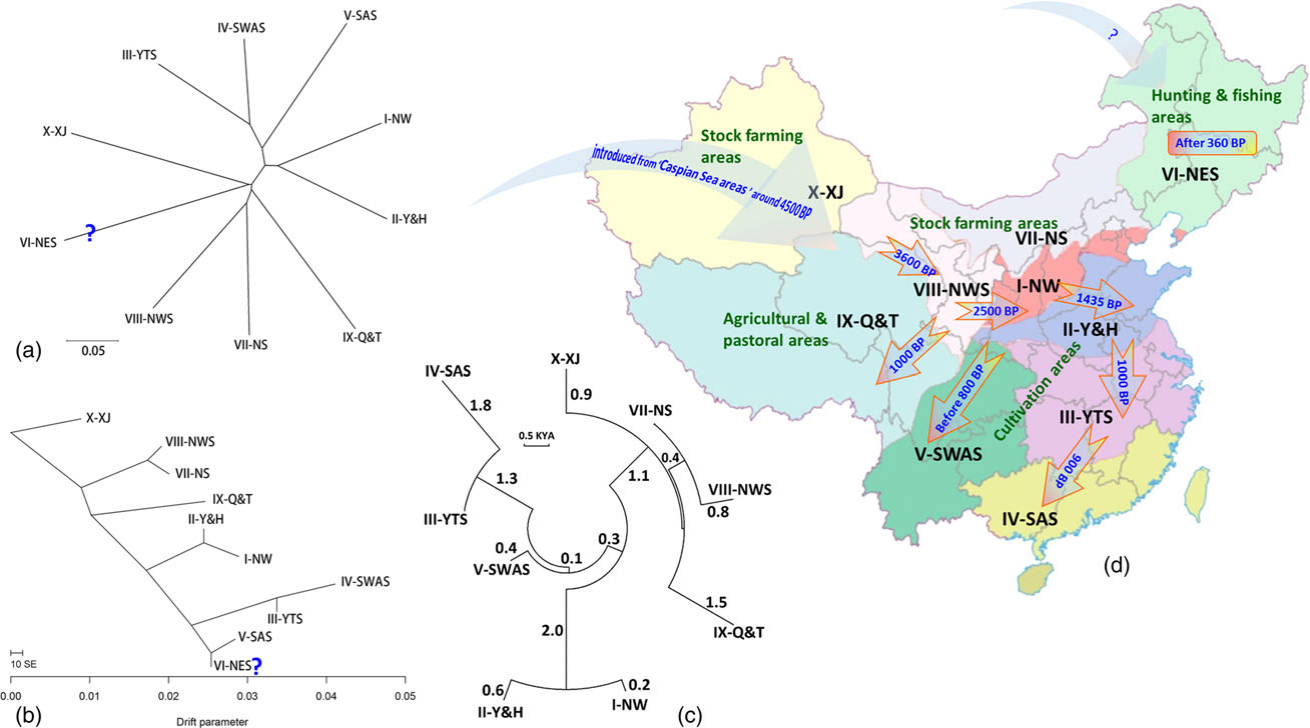
A neighbour‐joining tree (a) and a TreeMix tree (b) showing relationships among populations of landraces in 10 Chinese agro‐ecological zones. Unrooted NJ networks illustrating divergence time (TF) in generations during the distribution of wheat cultivation across China (c). TF was estimated using information from genetic distance calculated by 7001 DArT markers. Branch lengths are proportional to divergence times in thousands of years ago (KYA). The time and path of wheat distribution in China based on historical records (d). Wheat growing began in the VI‐NES area around 370 BP when soldiers brought it from Russia to Shengjing/Shengyang (He, 2008), thus there is an additional arrow from Russia.

The structure of genetic diversity was to a large extent consistent with the geographic distribution of Chinese wheat landraces. Landraces from northern zones VIII‐NWS and VII‐NS, I‐NW and II‐Y&H clustered together as did those from southern zones III‐YTS, IV‐SAS and V‐SWAS (Figure 4a,b). However, the landraces from a northeast zone, VI‐NES, did not show a clear relationship within the group, either in the NJ tree (Figure 4a) or in TreeMix (Figure 4b). The trees clearly separated the agro‐ecological zones as distinct clusters (Figure 4a,b). In this work, the history of the dispersion of wheat between zones could be inferred through estimates of T_F_ among zones. Wheat cultivation was first started in zones that were largely centred on animal husbandry (stock farming) in northwestern China and from there to areas in which agricultural production centred on field crop agriculture: central and southwestern China (Figure 4a–c). A large divergence time (about 5.5 KYA or 5500 generations) between landrace populations in X‐XJ (northwestern China) and those in IV‐SAS (southern China) is consistent with this scenario. This scenario also agrees with the idea of a single major introduction of wheat cultivation to China, the time of its arrival, and its subsequent spread throughout China based on historical records (Figure 4c,d).

### Evidence for selection

**Figure 5:**
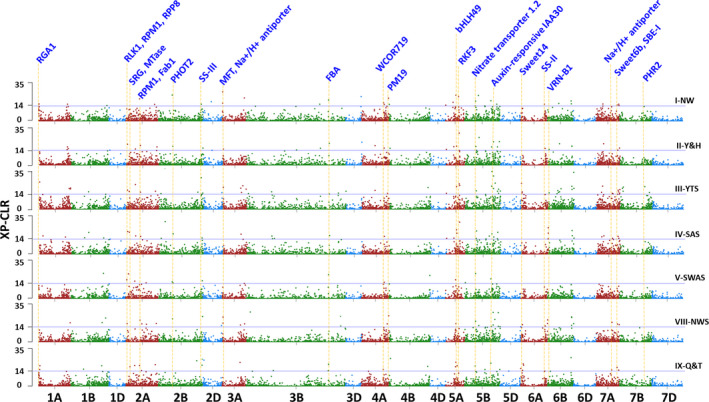
Chromosome regions under selection identified by XP‐CLR statistics for seven wheat growing zones. XP‐CLR scores along chromosomes of each zones were compared with those from Iran/Turkey. The horizontal line indicates a 1.5% genome‐wide cut‐off level. The positions of some important genes associated with seed dormancy, biotic/abiotic resistance, and starch was listed on the top. Details about the selected regions and candidate genes were listed in Table S7.

Evidence of selection was only investigated in wheat landrace populations in seven agro‐ecological zones, in which the number of landraces were adequate. To identify genomic regions most affected by selection, the XP‐CLR analysis software was used to scan groups of linked genes (Figure 5; Table S8A). Genomic regions with high XP‐CLR values indicate selection events. One or more genes present in such a region could have been a selection target (Table S8A). Using this technique, footprints of selection were detected in 148 genomic regions spanning 1.5% of the wheat genome. An average region contained nine annotated genes (Table S8A), and the mean strength of selection *s* was 0.1369, slightly higher than the 0.1101 found across the rest of the genome (*F* = 57.2, *P* < 0.005).

The numbers of selected regions differ among the three genomes and individual chromosomes. Of the 148 genomic regions showing signs of selection, seven (4.7%) were in the D genome, 69 (46.6%) were in the A genome, and 72 (48.6%) in the B genome. There were 12 or more selected regions in chromosomes 1A, 2A, 3B, 5B, 6A and 6B, while none were in 4D, 6D or 7D (Table S8B). Selection footprints were sometimes found in corresponding, presumably orthologous genes, and also in regions in homoeologous chromosomes. One such region was on chromosomes 2A at 107.5–108 Mb and 2B at 106–106.5 Mb. A majority of selected regions were in distal regions of chromosomes, except for chromosomes 3A, 4A, 5A, 5B, 6B and 7A (Figure 5). Among the 148 genomic regions showing signs of past selection, 80 were in landraces in unique agro‐ecological zones, 44.6% of selection footprints were shared by landraces in two or more zones, and 11 were shared by landraces in more than five zones. One such selection footprint was at 203.5 Mb on chromosome 5B, which was shared in landraces in all agro‐ecological zones. The 5B genomic region contained genes for disease resistance protein RPP13, NAC domain‐containing protein 9, FEZ protein, secologanin synthase and several genes for proteins of unknown function (Table S8).

The numbers of selection footprints present in individual agro‐ecological zones paralleled the spread of wheat cultivation across China suggesting that genes were selected in one environment, fixed and then landraces containing these genes were disseminated to the subsequent zone into which wheat cultivation was introduced (Figure 5; Table S8). This is shown in region V‐SWAS, one of the newest wheat growing zones in China, and landraces in this zone show the fewest genomic regions under selection. Moreover, 12 of the 16 regions under selection in V‐SWAS were already selected in other zones, in which wheat cultivation was historically earlier than that in V‐SWAS (Figure 4; Table S8). Conversely, 32 of 42 genomic regions under selection in zone VIII‐NWS, one of the oldest wheat growing zones, were fixed in the landraces in the growing zones into which wheat was subsequently dispersed (Figure 5; Table S8).

**Figure 6:**
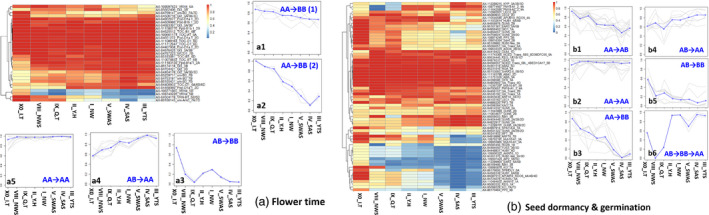
The major allele frequencies of SNPs in interdependent genes associated with flowering time (a), seed dormancy and germination (b) from Chinese wheat agro‐ecological zones gradually increased from about 0.5 to 1.0 or decreased from about 1.0 to 0.5 along the direction of wheat spread from northern west to southern China. AA was homozygous A allele, AB was heterozygous and BB was homozygous B allele. We defined the major allele as A, and the minor allele as B.

Major allele frequency (MAF) was used as an indicator of the genetic responses of wheat to local environments as wheat cultivation was dispersing from the Caspian Sea area to China. Flowering time is an important domestication/adaption trait that was altered during this dispersion. Wheat varieties with a winter growth habit require exposures to low temperatures (vernalization) to flower. Flowering time in wheat is primarily controlled by variation in VRN genes (Kippes *et al*., 2015) and genes controlling photoperiod insensitivity, Ppd genes (Laurie, 1997; Turner *et al*., 2005). MAF at *VRN*,* Ppd* and other genes associated with flowering timing gradually increased from about 0.5 to 1.0 or decreased from about 1.0 to 0.5 along the direction of wheat spread from colder zones I‐NW, II_Y.H, VIII‐NWS and IX‐Q&T in northern China to warmer zones III‐YTS, IV‐SAS and V‐SWAS in southern China (Figure 6a; Table S9A). The SNPs in dormancy and germination‐related genes could be clustered into six groups based on allele frequencies (Figure 6b; Table S9B). The minor allele frequencies of SNPs in groups b1 (Figure 6b.b1) and b4 (Figure 6b.b4) increased along with the spread of wheat, which is consistent with the rain and humidity trends in China (Figure 1). The frequency of SNPs in groups b2, b3 and b6 tend to progress from a polymorphic state in Iran/Turkey to a fixed, monomorphic state in northwestern and then southern China. In contrast, there were many genomic regions containing genes that have been identified as potentially important domestication genes, such as those in group b5, where allelic frequencies of SNPs did not show any significant change in the different environments, suggesting that those areas may have been under purifying selection. Examples of such genes are the ABA regulatory pathway, such as AIP2 (Gao *et al*., 2014), GUN5 (Somyong *et al*., 2011), ABI1 (Gosti *et al*., 1999) and ARF10 (Liu *et al*., 2007) in southern China zones III‐YTS, IV‐SAS and V‐SWAS. The seed dormancy‐related genes MFT (Nakamura *et al*., 2011) and PM19 (Barrero *et al*., 2015) have different allele frequencies among the growing zones. The landraces from southern zones show similar allele frequencies. Landraces from zones I‐NW and II‐Y&H in Central China show moderate variation in allele frequencies in genomic regions containing dormancy/germination‐related genes (Figure 6b).

## Discussion

### Genetic diversity of Chinese wheat landraces

In cereals, gene content and meiotic recombination is close to zero in the centromeric regions and increases as we move along the chromosome towards the telomeres for all chromosomes (Akhunov *et al*., 2003; Bauer *et al*., 2013; Chen *et al*., 2002; Mascher *et al*., 2017). The level of genetic variation is positively associated with the rate of recombination. In this study, genetic variation in Chinese wheat landraces is much higher at the telomeres than near the centromeres. This is consistent with data obtained from worldwide wheat germplasm, where a nonrandom variant distribution along the chromosomes was detected, with reduced variation near the centromeres and elevated variation at the telomeres (Akhunov *et al*., 2010; Choulet *et al*., 2014; Jordan *et al*., 2015). The differences in nucleotide diversity detected among the A, B and D genomes are similar between Chinese wheat landraces and worldwide hexaploid wheat (Akhunov *et al*., 2010; Jordan *et al*., 2015). Tajima's D and π for each genome obtained using Wheat660K data (Figures 2, S3) were also consistent with previous research using genotyping‐by‐sequencing (GBS) data (Jordan *et al*., 2015). However, the D genome had less nucleotide diversity and a lower divergence frequency than the A and B genomes (Figures 2, S3), consistent with the hypothesis that only a few *Aegilops tauschii* accessions from a small population contributing to the D genome of hexaploid wheat compared with the tetraploid ancestral donors of the A and B genomes (about 500 000 BP) (Wang *et al*., 2013), and its late addition (about 8000 BP) to hexaploid wheat (IWGSC, 2014) (Figures 2, S2).

### The spread of wheat in China

Agriculture originated in the Near East, where most crops were brought into domestication (Salamini *et al*., 2002), and major crops were then exported to other places. The expansion of the human environment out of centres of domestication into new latitudes presented plant communities with acute adaptive challenges (Allaby *et al*., 2015a). The adaptation of crops to certain environmental and climatic conditions may have contributed to the timing of agricultural spread (Jones *et al*., 2012). Two scenarios have been presented for the origins of wheat in China and potential pathways for its introduction: (i) wheat came from the West (eastward spread) through Central Asia, Afghanistan and Xinjiang (X‐XJ), along the ancient Silk Road; or (ii) wheat came from the northwest through Eurasia, southern Siberia and Mongolia, along the Eurasian Steppe route (Betts *et al*., 2014; Zhao, 2009). The first hypothesis is widely accepted based on paleobotanical evidence, genetic data and some archaeological analysis. However, several questions remain to be addressed.

The adaptation of plants to different environments is dynamic, presenting plants with new challenges leads to new adaptations, as also happens with changing agrarian practices (Fuller *et al*., 2010). For example, the *PPD‐H1* gene and simple sequence repeats (SSRs) in barley landraces have been used as evidence for environmental adaptation with agricultural spread in Neolithic Europe (Jones *et al*., 2012). Our population genetics results, based on both NJ tree and TreeMix data, show that most of the genetically related wheat landraces tend to have close geographic origins and that the population is structured along geographic lines (Figure 4), which was similar to previous analyses of wild emmer wheat (Nevo *et al*., 1991). The genetic relationship among landraces within wheat growing zones is consistent with the historical processes of wheat colonization across various Chinese wheat growing zones (Figure 4). Our genetic data indicate that Chinese wheat was imported via northwestern China, which is consistent with historical records. Wheat then moved to Central China (I‐NW) during the Shang dynasty (3600 BP), to the Yellow and Huai River valleys (west of II‐Y&H) during the Chunqiu and Han dynasties (2786–2218 BP), to Shandong (east of II‐Y&H) during the Sui and Tang dynasties (1435–1109 BP) and to the Yangzhi River regions (III‐YTS) during the Song and Yuan dynasties (1056–648 BP). After the Ming dynasty (648 BP), wheat was cultivated throughout China (Betts *et al*., 2014; Zeng, 2005a,b; Zhuang, 2002).

The genetic distance between wheat landraces from IX‐Q&T (Tibetan plateau) and those from VIII‐NWS (northwestern China) is not very large, as would be expected for two growing zones that are geographically close to each other (Figure 4). Recently, large numbers of wheat and barley seeds have been found in mid‐second millennium BC stone‐cist graves at the Yan'erlong site in Sichuan, in wheat growing zone IX‐Q&T (Tang *et al*., 2012), only about 100 km from zone VIII‐NWS. However, the appearance of wheat grown in western Sichuan and the northeastern Tibetan plateau probably corresponds to a westward migration from V‐SWAS (southwestern China), rather than a southward migration from northwestern China (Guedes *et al*., 2014; Hou *et al*., 2010). The ability of wheat and barley to tolerate frost and their low latitude growing degree requirements facilitated their spread into the IX‐Q&T growing zone at the high‐altitude margins of western China (Guedes *et al*., 2015; Zeng *et al*., 2015). Data from the eastern Tibetan Plateau show that wheat rapidly became the staple crop shortly after its introduction (Guedes *et al*., 2015). Wheat cultivation appeared later in hunting and fishing areas and pastoral areas than in cultivation areas where cereals such as millet and rice had already been growing for several millennia (Figure 4d). Recent data from the Tibetan Plateau and its surrounds show that a transition from millet to wheat and barley agriculture took place during the second millennium BC (Guedes *et al*., 2015). The data in this study show that the earliest wheat in this area was not imported from zone V‐SWAS. It should be noted that we analysed wheat landraces from the Tibetan Plateau rather than Tibetan semi‐wild wheat (*T. aestivum* ssp. *tibetanum* Shao).

In Figure 4, we show that the relationship of wheat landraces from VI‐NES (northeastern China) to the other landraces is not clear. In terms of genetic distance, VI‐NES is close to X‐XJ (Xinjiang) according to the NJ tree (Figure 4a) and close to IV‐SAS (South China) according to TreeMix data (Figure 4b), which is not consistent with a simple eco‐geographical dispersion process. There was no wheat in northeastern China (VI‐NES) before the Qing dynasty (372 BP), and wheat cultivation began in this area around 370 BP when soldiers brought it from Russia to Shengjing/Shengyang (He, 2008). This may be the reason that landraces from these areas are different from other Chinese wheat landraces. These data could provide additional insights into fine‐scale patterns of ancestry from wheat dispersion in China.

### Adaptation and selection of wheat landraces

Wheat has adapted to a wide range of climates, where the environment can affect genetic selection leading to genetic variation. The environmental factors of altitude, frost‐free days, annual sunlight, temperature and precipitation explain a significant proportion of the diversity, suggesting that natural selection may have produced regional/local divergence in Chinese landrace wheat as it has in wild emmer wheat (Ren *et al*., 2013). Therefore, natural selection appears to play an important role in phenological adaptation in landrace wheat, especially with a wide range of weather conditions from northern to southern China. Selective history and population genetic parameters interact with the available genetic variation in complex ways to shape the genetic architecture of a trait (Walsh, 2003). In cereals, most important agronomic traits are controlled by several genes, quantitative trait loci or complex pathways. For a polygenic trait, moderate or weak selection allows a greater long‐term response to selection than does very strong selection (Hamblin *et al*., 2011).

Selection during wheat improvement is weaker than that during domestication (Cavanagh *et al*., 2013; Gegas *et al*., 2010). Large amounts of change associated with a relatively small selective value can be characterized by using high‐throughput marker data (Allaby *et al*., 2015a). Identification of regions under selection can help us to understand the microevolution, distribution and selection of cereals, and could accelerate crop improvement in the future. Regions under selection can be detected using phenotypes that provide distinct information about positive selection, and combining the data from phenotypic analyses gives greater power for localizing the source of selection (Grossman *et al*., 2010). However, the effects of positive selection may have been amplified by population expansion, which followed wheat origin by hybridization of a limited number of *Ae. tauschii* and *T. turgidum* genotypes (Appels and Lagudah, 1990; Wang *et al*., 2013).

Phenotypic features of wheat landraces have been selected under different environmental and agricultural conditions, resulting in significant variation among populations. Furthermore, the lack of shared genomic regions might be because the genotypes underwent different selection pressures to adapt to local agricultural conditions, or the same agronomic trait may be obtained by selection on different genomic regions (Jiao *et al*., 2012). Limited sharing of coselected regions among genotypes from different geographic regions has also been observed in humans and maize (Jiao *et al*., 2012; Pickrell *et al*., 2009). Comparing selective sweeps identified in different populations could pinpoint selection that acts on distinct targets or multiple functionally equivalent alleles in different portions of the geographic range of wheat (Cavanagh *et al*., 2013). For example, the region containing PM19, a seed dormancy‐associated protein that is induced by ABA in wheat (Barrero *et al*., 2015), was under selection pressure in wheat landrace growing zones I‐NW, II‐Y&H, III‐YTS and VIII‐NWS, the earliest wheat cultivation areas (Figures 1, 5). Starch synthase II was under selection pressure in western China zones VIII‐NWS and IX‐Q&T, while the chromosome region containing starch synthase III had strong selection pressure in growing zone V‐SWAS (Figure 5). A region containing the cold stress associated gene WCOR719 was under strong selection in zone IX‐Q&T, which is one of the coldest zones in China (Figures 1, 5). We also found that some domestication regions (such as VRN gene regions on chromosome 6B) showed evidence of continual selection, indicating that these loci may contribute to phenotypes of sustained agronomic importance. The frequencies of VRN1 through VRN4 in landraces from different localities in East Asia correlated with the degree of winter coldness (Iwaki *et al*., 2000). Particular combinations of VRN1 and VRN3 alleles and photoperiod haplotypes show early‐maturing characteristics in wheat cultivars in these geographic regions in China (Chen *et al*., 2013). Additionally, the genes linked to these regions have also been affected by selection, limiting the diversity available for modern improvement (selective sweep). The selective sweep regions containing genes also associated with important agronomic traits and biotic/abiotic resistance including phototropin‐2 (*PHOT2*), vernalization (*VRN*), cold/freezing stress resistance (*WCOR719*), seed dormancy and germination (*MFT*,* PM19*), starch (*SS‐II*,* SS‐III*,* SBE‐I*), rust resistance (*RLK1*,* RPM1*,* RPP8*), salt response (*SRG*, Na+/H+ antiporter), sugar transport (Sweet14, Sweet6b) and others (Table S8; Figure 5).

Only a small fraction of the homoeologous regions harbouring selected variants have been found to overlap among wheat genomes in any given wheat line (Jordan *et al*., 2015). Evidence suggests that directional selection in allopolyploids rarely acts on multiple parallel advantageous mutations across homoeologous regions, likely indicating that a fitness benefit could be obtained by a mutation at any one of the homoeologous loci (Jordan *et al*., 2015). In this study, we rarely detected directional selection on the same gene from the A, B and D genomes in Chinese wheat landraces. Only the allele frequencies of SNPs AX‐110992931 in *LEC2(LEAFY COTYLEDON2)*_2A and AX‐95080933 in *LEC2*_2D show similar patterns during the dispersion of the Chinese landraces (Figure 6b; Table S9B).

Previous studies of local adaptation show that there is a trend towards complex adaptation involving many loci, each under relatively weak selection (Allaby *et al*., 2015a) in the populations undergoing such selection. The more conventional view of selection on domestication syndrome or important agronomic traits (biotic/abiotic stress resistance, yield and quality), a single mutant subject to a selective sweep, would achieve less selection overall than that found. Furthermore, a selective sweep may result in leaving the population or species vulnerable because of an inability to cope with further adaptive challenges (Allaby *et al*., 2015c). Thus, the idea that multiple loci controlling adaptive traits is targeted weakly to effect strong selection results rather than the more conventional viewpoint of a strong selective sweep at a single locus better fits our results (Figure 6; Table S9). It is therefore predicted that we should see multiple changes in the interactions of genes and their products, such as in regulatory and metabolic networks (Allaby *et al*., 2015b). Based on high‐throughput data, selection acting on genes that are interdependent in networks of interaction could be analysed. At a systems level, the majority of adaptation is expected to be achieved through the regulation of genes in networks, affecting rapid and complex responses to environmental stimuli (Alon, 2007). In wheat, we investigated details of the variance of allele frequencies for genes involved in seed dormancy/germination and flowering time (Figure 6), and it was clear that some SNPs in genes associated with the same traits showed similar tendencies, rather than only single genes being affected. For instance, the MAF of SNPs in genes VRN4, GI3, VRN3, GI2 and TOC1 was gradually changed along the path of the distribution of wheat from the eastern Mediterranean region to China (Figure 6a; Table S9A). Wheat is a facultative long‐day plant, adapted to short growing seasons, with long days promoting flowering in spring, while short days delay reproductive development (Hill and Li, 2016). During breeding and phenotypic selection, genetic variants at flowering loci were selected to optimize flowering time within a given production environment to achieve greater yields (Hill and Li, 2016; Jung and Müller, 2009). Also, the MAF of SNPs in genes *AGO4*,* MFT*,* Myb10‐A1*,* Sdr4* and *SPATULA* that affect seed dormancy and germination clearly increased along with the spread of wheat from the xeric northwest to the mesic south in China (Figure 6b; Table S9B). This result is consistent with other study data obtained from 781 Chinese wheat cultivars over the last 50 years (Xiao, 2004). Chinese wheat cultivars obtained by modern breeding processes utilizing landraces in zones I‐NW and II‐Y&H have no preharvest sprouting resistance, while cultivars bred from landraces from zone III‐YTS and V‐SWAS have high preharvest sprouting resistance levels (Xiao, 2004; Xiao *et al*., 2002). The frequency variations among Chinese wheat landraces utilized to breed modern Chinese wheat cultivars provide data to study microevolution events in cereal crops. Our data indicate that selection on important domestication or agronomic traits according to interdependent functional genes in their regulatory and metabolic networks has given wheat the ability to cope with adaptive challenges under complex environments from northwestern to southern China.

## Experimental procedures

### Chinese wheat landrace materials

In total, 717 wheat landraces were selected from ten Chinese agro‐ecological zones (He *et al*., 2001): I‐NW (northern winter wheat zone), II‐Y&H (Yellow and Huai River valleys facultative wheat zone), III‐YTS (middle and low Yangtze valleys autumn‐sown spring wheat zone), IV‐SAS (southern autumn‐sown spring wheat zone), V‐SWAS (southwestern autumn‐sown spring wheat zone), VI‐NES (northeastern spring wheat zone), VII‐NS (northern spring wheat zone), VIII‐NWS (northwestern spring wheat zone), IX‐Q&T (Qinghai–Tibetan plateau spring‐winter wheat zone) and X‐XJ (xinjiang winter–spring wheat zone). These landraces were planted in China before the 1950s. They were obtained from the Chinese Crop Germplasm Information System (http://icgr.caas.net.cn/cgris_english.html) and CAAS (Chinese Academy of Agricultural Science).

A total of 717 landraces were genotyped by the DArTseq (http://www.diversityarrays.com/) platform, and 272 landraces and 13 Iranian and Turkish wheat landraces were genotyped using an Affymetrix Axiom Wheat660K single‐nucleotide polymorphism (SNP) array (designed by Jizeng Jia, Chinese Academy of Agriculture Sciences and synthesized by Affymetrix (http://wheat.pw.usda.gov/ggpages/topics/Wheat660_SNP_array_developed_by_CAAS.pdf) (Figure 1; Table S1). This strategy covered most regions of the wheat genome with markers in a cost‐effective manner.

### Markers and their alignment to the Chinese Spring survey sequence and *Ae. tauschii* genome sequence

The chromosome locations of 89 284 DArTseq markers (DArT and DArT_GBS) and 630 517 SNP markers from the Wheat660K array were determined by alignments of their sequences to the genomic sequence of common wheat, cultivar Chinese Spring, popseq.28.dna (ftp://ftp.ensemblgenomes.org/pub/plants/release-28/fasta/).

The following database and blast commands were used to locate the physical location of probes (markers): blastdb ‐max_file_sz 20G ‐dbtype nucl ‐in / IWGSC2.28/database/IWGSC2.28 ‐out / /database/Triticum_urartu.GCA_000347455.1.28.dna.toplevel and blastall ‐p blastn ‐e 1e‐10 ‐b 1 ‐v 1 ‐m 8 ‐i /seq/Wheat660(DArT)_probes.fa ‐o / blastn/ Wheat660(DArT)_blast1 ‐d /IWGSC2.28/database/IWGSC2.28.

In total, 2 060 458 sequences were obtained from the IWGSC database and blasted against the Wheat660K SNP marker probes, and 85 253 and 22 367 sequences against DArT and DArT_GBS probes, respectively. SNP and DArT probes matching multiple locations were removed. In addition, markers with more than 25% missing data were deleted. Those with <5% mismatched nucleotides and with identity > 95% were selected. This generated a physical map containing 27 933 markers detectable by DArTseq and 312 831 markers detectable by Wheat660K SNPs (Table S2). Marker density along chromosomes was analysed using PowerMarker V3.25 (Liu and Muse, 2005), in which the marker density was calculated using 500‐kb windows.

In the next step, markers with >10% missing values and minor allele frequency < 0.05 were discarded. The *Ae. tauschii* reference sequence (http://aegilops.wheat.ucdavis.edu/jbrowse/index.html?data=Aet%2Fdata%2F&loc) was employed to determine the locations of the remaining markers in the D genome, using the same strategy as that used for locating the chromosome positions of SNP markers in the Chinese Spring genomic survey sequences. One SNP marker per Mb of the *Ae. tauschii* pseudomolecule was selected to anchor the locations of markers along wheat D chromosomes. Subsequent analyses of nucleotide diversity (π) and the Watterson estimator (θ) were also carried out. The genetic variance among wheat growing zones was analysed with ANOVA.

### Population genetic analysis and genome scanning for selection signals

Structure (Pritchard *et al*., 2000, http://pritchardlab.stanford.edu/software.html) was used to infer population structure using an admixture model, ten replicates at each *K* value and 100 000 iterations of burn‐in followed by 100 100 MCMC iterations. A model‐based method was used to estimate the population structures of Chinese wheat landraces. The potential number of populations, *K*, was estimated using the LnP(D) (Pritchard *et al*., 2000) and ∆*K* (Evanno *et al*., 2005) statistics in a trial of 10 individual runs for *K* ranging from 1 to 9. Variance of LnP(D) and the mean of LnP(D) indicated a hierarchical structure for Ks from 2 to 5. Increase in *K* beyond 6 led to lower likelihoods and higher variance among runs, which was accompanied by unstable clustering of the landraces. Results of independent runs were analysed, and figures were drawn using R. A neighbour‐joining (NJ) tree was constructed using MEGA 5.0 (Tamura *et al*., 2011) based on genetic distances between wheat growing zones calculated in R.

The software TreeMix (http://pritchardlab.stanford.edu/software.html) was used for estimation of phylogeny with admixture. Divergence time (TF) in generations was estimated by TF = 2Ne*F*_ST_. *F*_ST_ is the proportion of the variance in allele frequencies that is found between subpopulations (Holsinger and Weir, 2009). In the absence of selection and migration, *F*_ST_ between populations is essentially governed by genetic drift. A total 7001 DArTseq markers chosen randomly with a missing rate <20% were used to calculate *F*_ST_ in Arlequin ver 3.1 (Excoffier *et al*., 2005). The impact of genetic drift is largely determined by current effective population size (*N*_e_) in populations and divergence time (*T*) as *F*_ST_ = *T*/2*N*_e_ (Nei, 1987). The current effective population size (*N*_e_) was estimated using multilocus diploid genotypes from population samples by NeEstimator V2 (Do *et al*., 2014). The genetic relationships among landraces from different growing zones based on *N*_e_ and *F*_ST_ were compared with historical records.

The following population statistics were computed in 500‐kb windows sliding by 50‐kb. The genetic diversity of landraces from the seven main growing zones was analysed with Variscan (Hutter *et al*., 2006) for each window using Tajima's D, nucleotide diversity (π) (Tajima, 1983) and Waterson's estimator of nucleotide diversity (θ) (Watterson, 1975). Theta = 4*N*μ, where *N* is the effective population size, and μ is the mutation rate per nucleotide (or per sequence) per generation.

Each of the 500‐kb windows was considered a selection window in a Chinese agro‐ecological zone if it were in the top 1.5% of all 500‐kb windows of empirical distribution for XP‐CLR values. Each 500‐Kb region matching this rule was considered a ‘sweep window’, and using gene annotations, the numbers and type of candidate genes were calculated for each sweep window. In addition, to identify signals of selection, we performed a genome scan using an updated cross‐population composite likelihood approach in XP‐CLR version 1.0 (Chen *et al*., 2010). Evidence for selection across the genome in the Chinese agro‐ecological zones, and the Fertile Crescent gene pool was evaluated as follows. A 500‐kb sliding window with 50‐kb steps across the whole genome was used for scanning. The command used was ‘XPCLR –c freqInput output –w1 gWin (Morgan) snpWin gridSize (bp) chrX –p0 0.95’.

### Environmental factors and geographical genetic analysis of landraces

The environmental factors of wheat agro‐ecological zones in China were collected from the book ‘Chinese Wheat’ (Jin, 1996) and integrated with data from the Data Center for Resources and Environmental Sciences, Chinese Academy of Sciences (RESDC) (http://www.resdc.cn). In the absence of data on ancient environmental conditions in each zone, recent environmental history was assumed to approximate environmental conditions prevailing in the past. Monthly measurements of precipitation for the last 100 years and air temperature for the last 50 years were downloaded from about 630 stations throughout China and used to analyse the intersection of the geographical distribution with the genetics of Chinese wheat landraces.

The genetic diversity of the seven main wheat growing zones (I‐NW, II‐Y&H, III‐YTS, IV‐SAS, V‐SWAS, VIII‐NWS and IX‐Q&T) was examined using the methods described earlier. Observed precipitation and air temperature for each month during the wheat growing season for the main zones with sufficient environmental and genetic index data were computed (Table S3). Multiple regression analysis was performed in R to investigate the relationship between population diversity and the following environmental variables: altitude (Al), number of frost‐free days (Fd), annual sunlight (As), accumulated temperature (At) and precipitation amount (Pa).

## Conflict of interest

The authors declare no conflict of interest.

## Authors’ contributions

Zhou Y carried out the experiment, analysed the data and contributed to writing for the population genetics section; Chen ZX analysed the data and contributed to writing for the population genetics section; Cheng MP analysed the data and prepared the figures for the manuscript; Chen J contributed to writing the spread and distribution parts of manuscript; Zhu TT contributed to genomic location of probes on *Aegilops tauschii*; Wang R participated in the analysis of population genetics data; Liu XY participated in the field experiment; Qi PF contributed to writing the functional genes selection parts of manuscript; Chen GY managed plant materials; Jiang QT participated in the field experiment; Wei YM participated in the field experiment; Luo MC carried out sequence analysis with the *Aegilops tauschii* genome; Nevo E contributed to the writing for the evolution and adaption of wheat section; Allaby RG contributed to the analysis and writing for the selection of wheat section; Liu DC contributed to the writing for the population genetics and selection section; Wang JR formulated the questions, designed and carried out the experiment, analysed the data and wrote the manuscript; Dvorak J contributed to the genomic and population data analysis and writing of the manuscript; Zheng YL participated in the design of the experiments.

## Supporting information


**Figure S1** Numbers of DArTseq markers (A) and Wheat660k SNP markers (B) based on CS survey popseq.28.dna per wheat genome and chromosome.
**Figure S2** Physical map of DArTseq and 660k_SNP markers based on CS genome. (a), density of 660k_SNPs in 100Kb windows; (b), density of DArTseq in 100Kb windows; (c), Heterozygosity (HeteFreq) of 660k_SNP markers; (d), HeteFreq of DArTseq markers; (e), PIC of 660k_SNP markers; (f), PIC of DArTseq markers.
**Figure S3** Comparison of the average nucleotide diversity (π) and Tajima's D on the A, B, and D genomes of Chinese wheat landraces from Wheat660K and DArTseq data with that of 62 worldwide wheat cultivars from whole exome capture and genotyping‐by‐sequencing data (Jordan *et al*., 2015).
**Figure S4** Distribution of 660k_SNP markers, π and Tajima's D along D‐genome chromosomes in wheat and *Ae. tauschii*. One marker per Mb on *Ae. tauschii* chromosomes was selected. Red lines connect corresponding markers on the wheat D genome chromosomes and homologous *Ae. tauschii* chromosomes.
**Figure S5** Comparison of the landraces from different wheat growing zones by nucleotide diversity (π) and theta (θ).
**Figure S6** Population structure of 285 landraces by wheat660K array (A); population structure of 717 landraces by DArTseq array (B); neighbour‐joining tree of 717 Chinese landraces genotyped by DArTseq (C); 285 landraces including 272 Chinese wheat and 13 Iran/Turkey wheat landraces (0‐I&T) genotyped by Wheat660 (D); comparison of landraces in neighbour‐joining tree and topological structure (E); distribution of the four main groups in wheat growing zones in China (F).

**Table S1** List of 730 Wheat landrace accessions used in this study and geographic origins. # = genotyping by DArT and * = genotyping by Wheat660K. The accessions belonged to the group in topological structure data when K = 5 was also listed in Wheat660_K5 and DArT_K5
**Table S2** Distribution of markers (based on CSS survey popseq.28.dna) by DArT, DArT‐seq and Wheat660K among various genomic features (A); the location of DArT and SNP markers on CS genomic survey sequences and *Ae. tauschii* chromosomes (B)
**Table S3** Data used for evaluating the relationship between climatic/geographic factors and genetic index
**Table S4** Genetic variance and genetic index of Chinese wheat landraces on each chromosome
**Table S5** The *F*_ST_ (upper) and genetic distance (down) of Chinese wheat landraces among major plant zones
**Table S6** The Pi and Theta of Chinese wheat landraces among major plant zones along the chromosomes
**Table S7** Coefficient of multiple regressions between genetic indices and environmental variables of landrace in Chinese major wheat growing areas
**Table S8** Selected regions detected by XP‐CLR in major wheat growing zones of China (A); Selected regions detected by XP‐CLR (>=14) in major wheat growing zones of China (B)
**Table S9** Allele frequency of SNPs in genes associated with flower time (A), seed dormancy and germination (B) from different Chinese wheat plant zones
