# Supplementary material for: Uncovering the dispersion history, adaptive evolution and selection of wheat in China
Source: Plant Biotechnol J. 2017 Jul 17;16(1):280–91. doi: 10.1111/pbi.12770 (PMC5785339; doi:10.1111/pbi.12770)
Supplement: Supplementary file 1 — Figure S1 Numbers of DArTseq markers (A) and Wheat660k SNP markers (B) based on CS survey popseq.28.dna per wheat genome and chromosome. Figure S2 Physical map of DArTseq and 660k_SNP markers based on CS genome. (a), density of 660k_SNPs in 100Kb windows; (b), density of DArTseq in 100Kb windows; (c), Heterozygosity (HeteFreq) of 660k_SNP markers; (d), HeteFreq of DArTseq markers; (e), PIC of 660k_SNP markers; (f), PIC of DArTseq markers. Figure S3 Comparison of the average nucleotide diversity (π) and Tajima's D on the A, B, and D genomes of Chinese wheat landraces from Wheat660K and DArTseq data with that of 62 worldwide wheat cultivars from whole exome capture and genotyping‐by‐sequencing data (Jordan et al., 2015). Figure S4 Distribution of 660k_SNP markers, π and Tajima's D along D‐genome chromosomes in wheat and Ae. tauschii. One marker per Mb on Ae. tauschii chromosomes was selected. Red lines connect corresponding markers on the wheat D genome chromosomes and homologous Ae. tauschii chromosomes. Figure S5 Comparison of the landraces from different wheat growing zones by nucleotide diversity (π) and theta (θ). Figure S6 Population structure of 285 landraces by wheat660K array (A); population structure of 717 landraces by DArTseq array (B); neighbour‐joining tree of 717 Chinese landraces genotyped by DArTseq (C); 285 landraces including 272 Chinese wheat and 13 Iran/Turkey wheat landraces (0‐I&T) genotyped by Wheat660 (D); comparison of landraces in neighbour‐joining tree and topological structure (E); distribution of the four main groups in wheat growing zones in China (F). [file PBI-16-280-s001.docx]

**
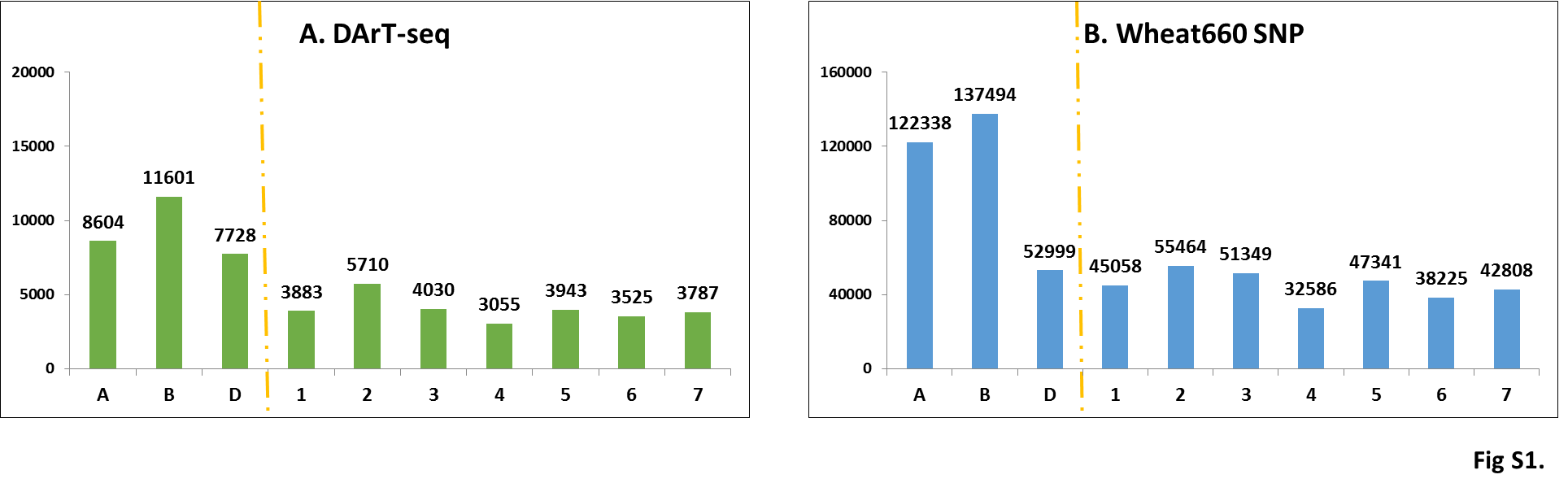
**

**Fig. S1** Numbers of DArTseq markers (A) and Wheat660k SNP markers (B) based on CS survey popseq.28.dna per wheat genome and chromosome.

**
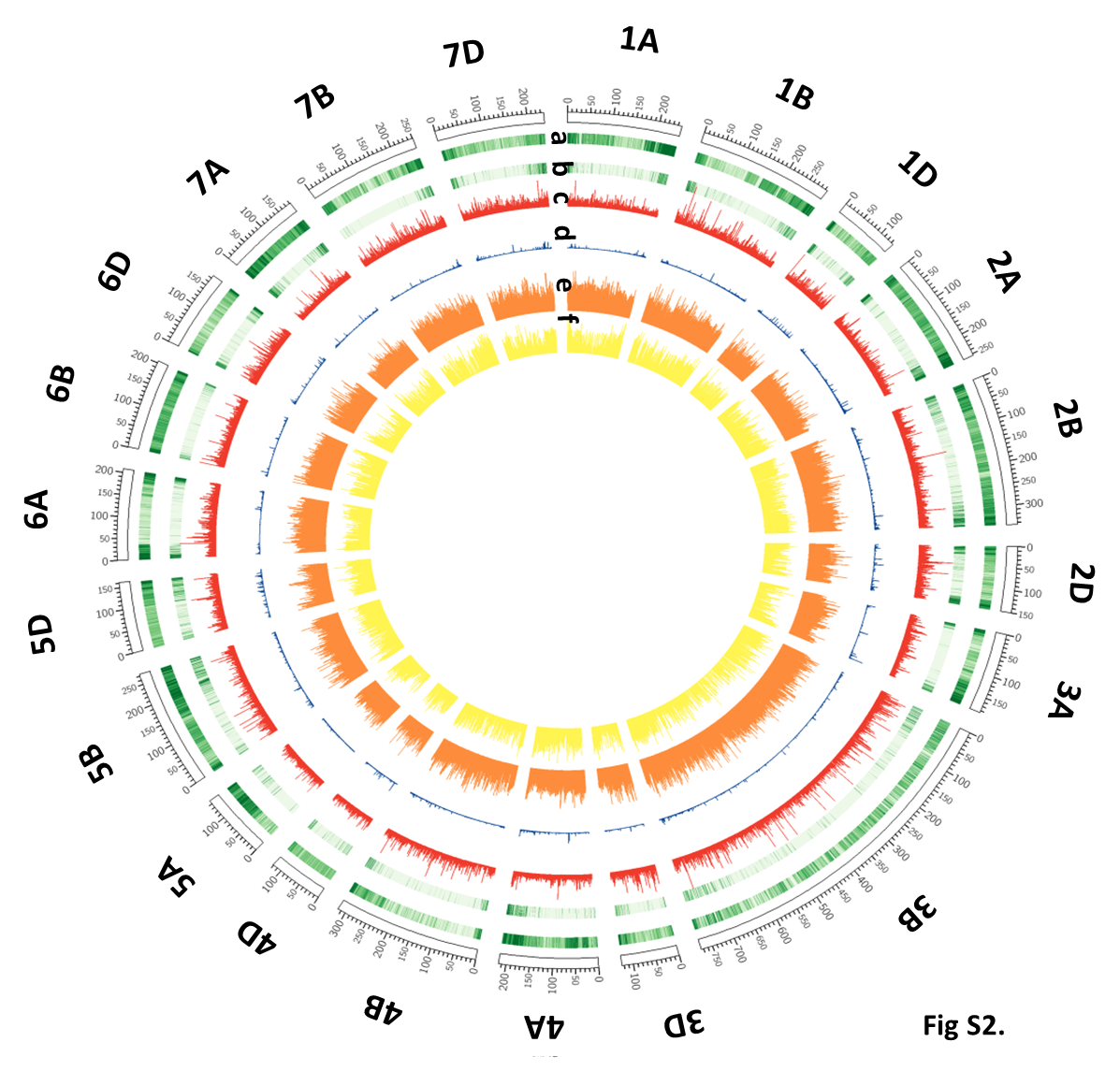
**

**Fig. S2** Physical map of DArTseq and 660k_SNP markers based on CS genome. (a), density of 660k_SNPs in 100Kb windows; (b), density of DArTseq in 100Kb windows; (c), Heterozygosity (HeteFreq) of 660k_SNP markers; (d), HeteFreq of DArTseq markers; (e), PIC of 660k_SNP markers; (f), PIC of DArTseq markers.

**
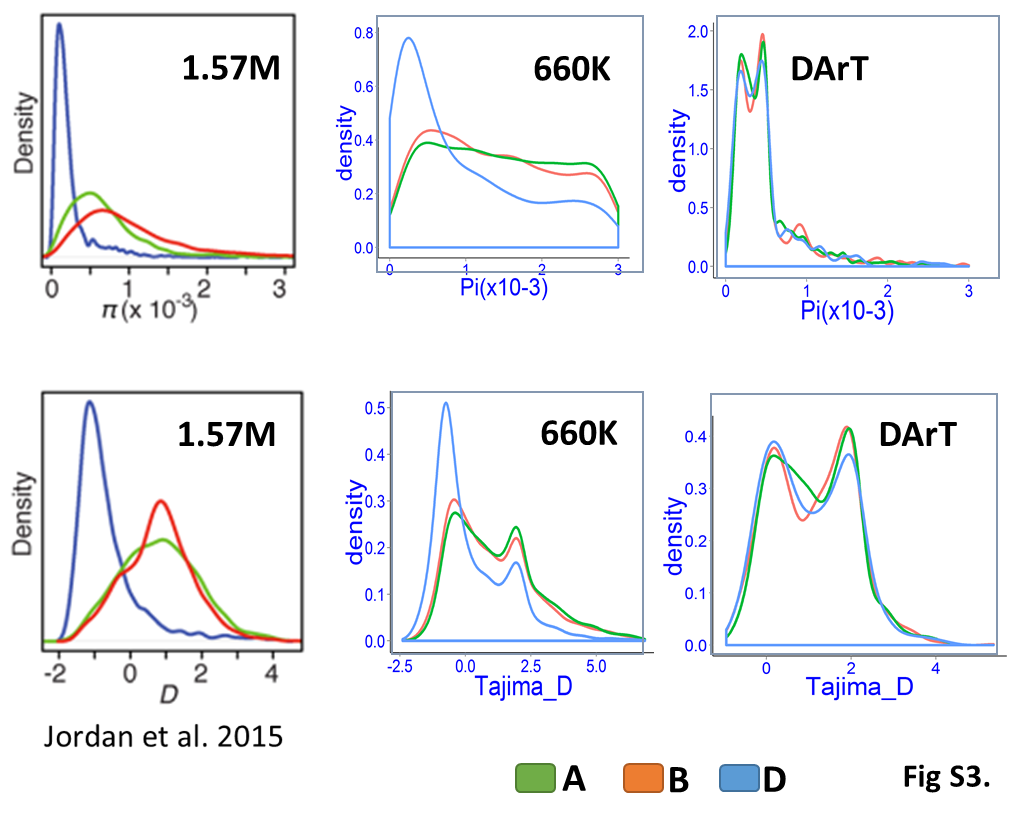
**

**Fig. S3** Comparison of the average nucleotide diversity (π) and Tajima’s D on the A, B, and D genomes of Chinese wheat landraces from Wheat660K and DArTseq data with that of 62 worldwide wheat cultivars from whole exome capture and genotyping-by-sequencing data (Jordan et al., 2015).


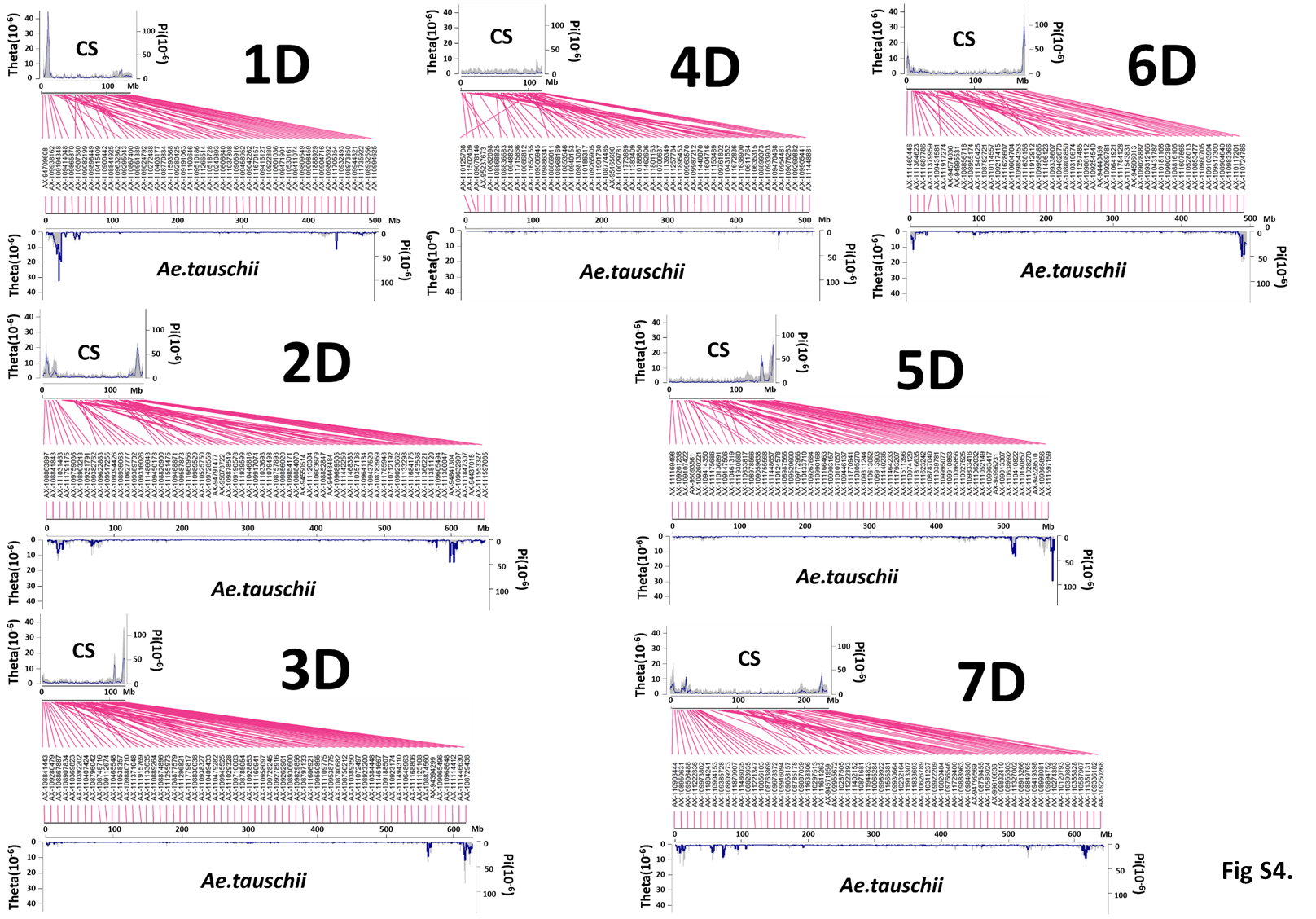


**Fig. S4** Distribution of 660k_SNP markers, π and Tajima’s D along D-genome chromosomes in wheat and *Ae. tauschii*. One marker per Mb on *Ae. tauschii* chromosomes was selected. Red lines connect corresponding markers on the wheat D genome chromosomes and homologous *Ae. tauschii* chromosomes.

**
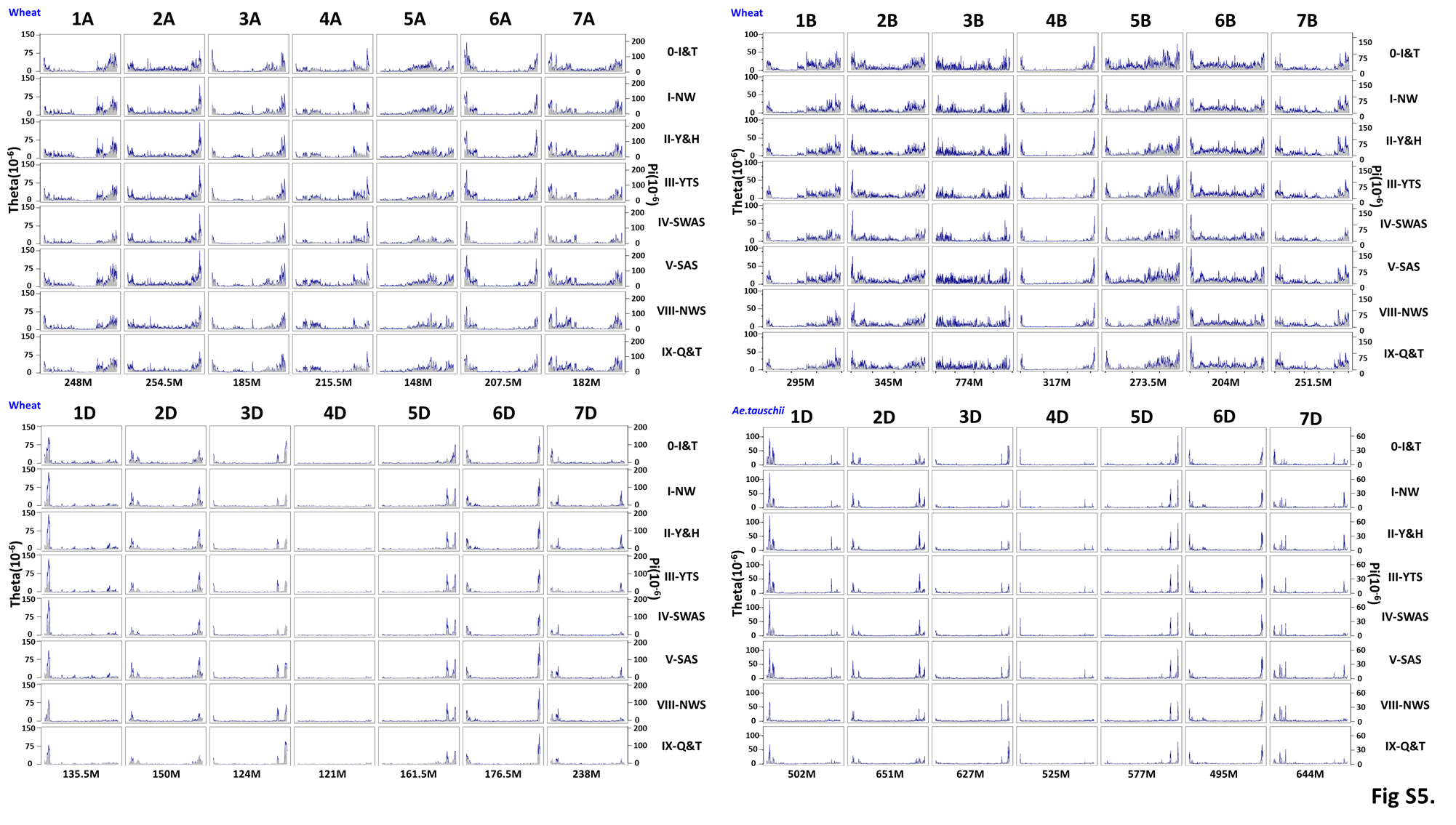
**

**Fig. S5** Comparison of the landraces from different wheat growing zones by nucleotide diversity (π) and theta (θ).

**
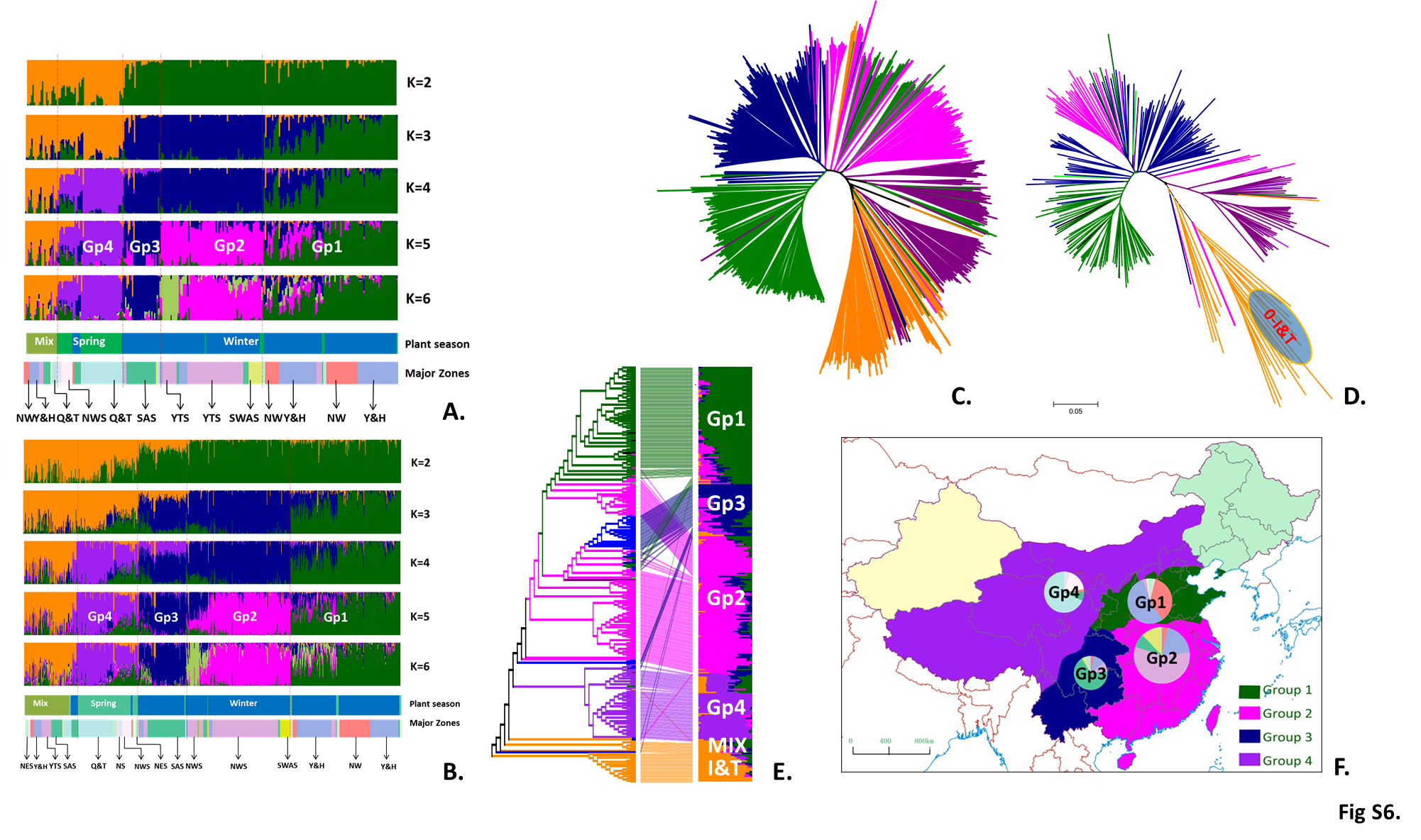
**

**Fig. S6** Population structure of 285 landraces by wheat660K array (A); population structure of 717 landraces by DArTseq array (B); neighbor-joining tree of 717 Chinese landraces genotyped by DArTseq (C); 285 landraces including 272 Chinese wheat and 13 Iran/Turkey wheat landraces (0-I&T) genotyped by Wheat660 (D); comparison of landraces in neighbor-joining tree and topological structure (E); distribution of the four main groups in wheat growing zones in China (F).
